# Evaluating the mortality and health rate caused by the PM_2.5_ pollutant in the air of several important Iranian cities and evaluating the effect of variables with a linear time series model

**DOI:** 10.1016/j.heliyon.2024.e27862

**Published:** 2024-03-13

**Authors:** Zahra Kazemi, Ahmad Jonidi Jafari, Mahdi Farzadkia, Payam Amini, Majid Kermani

**Affiliations:** a Research Center for Environmental Health Technology, Iran University of Medical Sciences, Tehran, Iran; b Department of Environmental Health Engineering, School of Public Health, Iran University of Medical Sciences, Tehran, Iran; c Department of Biostatistics, School of Health, Iran University of Medical Sciences, Tehran, Iran

**Keywords:** AirQ^+^ software, Mortality, Health effect, Air pollution

## Abstract

All over the world, the level of special air pollutants that have the potential to cause diseases is increasing. Although the relationship between exposure to air pollutants and mortality has been proven, the health risk assessment and prediction of these pollutants have a therapeutic role in protecting public health, and need more research. The purpose of this research is to evaluate the ill-health caused by PM_2.5_ pollution using AirQ ^+^ software and to evaluate the different effects on PM_2.5_ with time series linear modeling by R software version 4.1.3 in the cities of Arak, Esfahan, Ahvaz, Tabriz, Shiraz, Karaj and Mashhad during 2019–2020. The pollutant hours, meteorology, population and mortality information were calculated by the Environmental Protection Organization, Meteorological Organization, Statistics Organization and Statistics and Information Technology Center of the Ministry of Health, Treatment and Medical Education for 24 h of PM_2.5_ pollution with Excel software. In addition, having 24 h of PM_2.5_ pollutants and meteorology is used to the effect of variables on PM_2.5_ concentration. The results showed that the highest and lowest number of deaths due to natural deaths, ischemic heart disease (IHD), lung cancer (LC), chronic obstructive pulmonary disease (COPD), acute lower respiratory infection (ALRI) and stroke in The effect of disease with PM_2.5_ pollutant in Ahvaz and Arak cities was 7.39–12.32%, 14.6–17.29%, 16.48–8.39%, 10.43–18.91%, 12.21–22.79% and 14.6–18.54 % respectively. Another result of this research was the high mortality of the disease compared to the mortality of the nose. The analysis of the results showed that by reducing the pollutants in the cities of Karaj and Shiraz, there is a significant reduction in mortality and linear modeling provides a suitable method for air management planning.

## 1 Introduction

Air pollution is a global problem that has significant negative effects on the ecosystem and people's health [1]. Air pollutants are in the form of particles, liquid droplets, gases or a combination of them, which are very complex [2]. Exposure to these pollutants in the short and long term has harmful effects on people's health [[3], [4], [5]]. Numerous studies have also shown that exposure to these pollutants leads to a wide range of diseases and deaths [[6], [7], [8], [9], [10], [11], [12], [13], [14]]. Ischemic heart (IHD), lung cancer (LC), chronic obstructive pulmonary disease (COPD), acute lower respiratory infection (ALRI), stroke and short-term cardiovascular and respiratory hospitalizations are some of the problems of exposure to this pollutant. are those that entail very high costs for the society [13]. The World Health Organization (WHO) reported that about 80%, 14% and 6% of deaths from IHD and stroke, COPD and LC are related to outdoor air pollution [14]. Particulate matter (PM), nitrogen dioxide, carbon monoxide, sulfur dioxide, lead and ozone have been introduced as six standard pollutants that affect people's health [9,15]. PM_2.5_ is one of the dangerous and important atmospheric pollutants whose concentration is increasing with the increase of industrial activities [[16], [17], [18]]. These compounds are usually emitted from industrial activities, combustion of fossil and domestic fuels, traffic, cigarette smoke, and vehicles [19,20] and lead to a decrease in heart rate, increase in blood pressure, and disruption of cells. Endothelial vessels become [21]. Krewski et al. reported in their research that with an increase in PM_2.5_ pollutant concentration, mortality from lung, heart and lung cancer increases by 9%, 4% and 9%, respectively [22]. Li et al. also stated during their study that with an increase of 10 μg/m^3^ of PM_2.5_ concentration, the death rate due to cardiovascular diseases (CVD) increases by 39% [23]. Also, during the research conducted by Wu et al. in Langyu it was observed that with an increase of 10 μg/m^3^ in the concentration of PM_2.5_, the rate of ischemic heart disease, stroke and cardiovascular diseases increases by 0.61%, 1.22% and 0.27%, respectively [24].

AirQ^+^ software is the most reliable method to evaluate the negative effects of exposure to air pollutants on human health at a specific time and place [9], which uses information on air pollutants, the total population and the population at risk, the base incidence, risk The relative (RR) and other epidemiological data estimate natural mortality rates and deaths from ALRI, COPD, IHD, LC, and Stroke [25]. Since according to the announcement of the World Health Organization (WHO), the big cities of Iran have been introduced as the most polluted cities in the world [13] and with regard to the mentioned contents; Assessment of the adverse health effects of exposure to PM_2.5_ pollutants in the cities of Arak, Esfahan, Ahvaz, Tabriz, Shiraz, Karaj and Mashhad and prediction of PM_2.5_ concentration was done. This research is very necessary and important not only for maintaining people's health, but also for planning and developing guidelines, which has not been studied comprehensively so far. Therefore, according to the sensitivity of the subject and the few studies that have been conducted in this field, the purpose of this research is 1) to estimate the adverse health effects of exposure to PM_2.5_ pollutants 2) to the effect of variables on PM_2.5_ concentration in the air of 7 important cities of Iran during the years 2020 It is 2019.

## 2 Methodology

During this research, the adverse health effects were first evaluated using AirQ^+^ software in 7 cities of Iran (Figure s1), and then the effect of various variables on the PM_2.5_ concentration was evaluated using the linear time series model (Fig. 1).

**Fig. 1:**
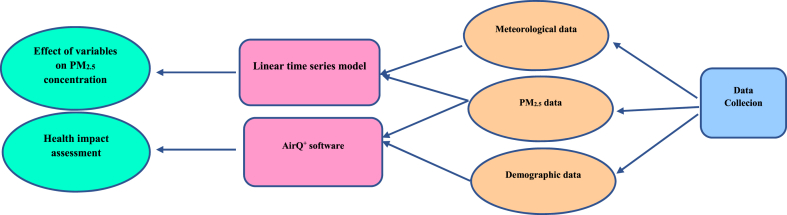
The performance of steps in this study.

### 2.1 Data gathering and processing

Hourly concentrations of PM_2.5_ pollutants from the comprehensive monitoring office of the country's environmental protection organization, population data from Iran's statistical center, meteorological data from the country's meteorological organization, and death statistics from the statistics and information technology center of the Ministry of Health, Medicine and.

Medical Education during The year 2019–2020 was received. Population information includes the total population, adults over 25 and 30 years old and children under 5 years old to estimate health effects and meteorological information including temperature (tmpf), relative humidity (relh), wind direction (drct), wind speed (sknt) and pressure (p01i) was After removing zero and negative PM_2.5_ pollutant data, the 24-h average pollutant concentration was calculated according to WHO standard with Excel software [26].

### 2.2 AirQ ^+^ software

AirQ^+^ is a software that is defined based on dose-response and baseline incidence (BI) and relative risk (RR) functions. Relative risk is the probability of contracting a disease as a result of exposure to atmospheric pollutants [27,28]. RR values of different deaths caused by exposure to PM_2.5_ pollutants and (BI according to WHO standard per 10^5^ people) are given in Table 1. These values were obtained from the AirQ^+^ software data file approved by the WHO European Center for Health and Environment [27,[29], [30], [31]]. Data required by the software; Annual average values of PM_2.5_ pollutant (μg/m^3^), population at risk (number or percentage), basic incidence per 10^5^ population and total population. Relative risk (RR) and Attributable Proportion (AP) are related to each other. The estimation of health effects in order to determine the health parameters of exposure to a specific atmospheric pollutant in a certain period of time and a certain population, was done based on the attributable population proportion [32,33]. The relationship between Attributable Proportion and Relative Risk is shown in Eqn (1).

(1) $$\;\text{AP}=\frac{\sum \left[\left(RR\left(c\right)-1\right)\times P\left(c\right)\right]}{\left[RR\left(c\right)\times P\left(c\right)\right]}\;$$

Here, the relative risk value for specific health impacts in category "c" of exposure (c)RR, which is obtained from the dose-response functions, AP attributable proportion of the health impacts, P(c) exposed group of the population proportion It is in category "c".

**Table 1:** Long-term health endpoints, baseline incidence (BI) range rates and relative risk (RR) values used in this study.

Air pollutant	Health endpoints	BI per 10^5^ capita							RR (CI)	RR references
		Arak	Esfahan	Ahvaz	Tabriz	Shiraz	Karaj	Mashhad		
**PM**_**2.5**_	Mortality, all-cause (age ≥30)	966.0	777.40	817.0	1003.0	797.0	549.0	936.4	1.062 (1.04–1.083)	[34]
	Mortality, COPD (age ≥30)	18.73	12.63	8.39	20.0	13.0	7.17	15.06	IER function	[34]
	Mortality, LC (age ≥30)	15.60	9.62	12.01	19.97	12.46	10.52	16.8	IER function	[34]
	Mortality, IHD (age ≥25)	157.45	125.13	155.06	98.18	107.0	131.33	114.0	IER function	[35]
	Mortality, Stroke (age ≥25)	85.46	53.05	61.5	48.0	62.77	31.42	65.93	IER function	[35]
	Mortality, ALRI (age ≤5)	13.24	23.89	14.09	20.0	15.21	11.28	11.34	IER function	[35]

The rate of attributable proportion caused by exposure to pollutants can be estimated when the baseline frequency of the specific health impact in the population is in the form of Eqn (2).

(2) IE=I×AP

Here I is the baseline incidence of the health effect in the study population, IE is the health impact rate attributable to the exposure [28,31,36].

In order to calculate the population, the number of estimated excess cases due to exposure was obtained using Eqn (3).

(3) NE=IE×N

NE is the number of excess cases, and N is the size of the population under study. Hypothetical RR values are included in AirQ^+^ software. The linear-log method was used to estimate the relative risks of pollutants.

### 2.3 Evaluation of the effect of different variables on the PM_2.5_ concentration using the time series linear model

In this study, the target variable (PM_2.5_) is a quantitative continuous variable that is recorded as a time series in different cities. In order to investigate the trend of the time series and also the effect of the variables, the linear model of the time series was used. This model is the application of the linear model in the time series data, in which it provides the possibility to examine the effect of the trend as well as the seasons of the time series in addition to other auxiliary variables on the target variable. The model is as follows Eqn (4):

(4) $${PM2.5}_{t}=\beta_{0}+\beta_{1}February+\ldots +\beta_{11}December+Trend+\beta_{12}X_{1}+\ldots +\varepsilon$$

in which the variable PM_2.5_ at time t was evaluated with the help of a width from the origin (β_0), seasonal variables (here monthly), time series trend and other variables investigated in the study with an error rate equal to ε.

### 2.4 Statistical analysis

Quantitative variables in this study are described as mean ± standard deviation. To make the time series model, the time series linear model has been used. The analyzes were done with the help of statistical programming software R version 4.1.3 and SPSS software version 20.

## 3 Results and discussion

### 3.1 PM_2.5_ pollutant concentration and meteorological parameters

The information obtained from the country's environmental organization and meteorological organization was used to estimate the health effects and predict the pollutant (PM_2.5_). Table 2 shows the average annual concentration of PM_2.5_ pollutant and meteorological parameters during 2019–2020. The highest and lowest average annual concentrations of PM_2.5_ with the values of 43/33 ± 19/23 and 24/12 ± 6/35 μg/m^3^ were related to Ahvaz and Arak cities, respectively. The World Health Organization declared the annual average of PM_2.5_ is less than 10 μg/m^3^ [37]. Therefore, the average annual concentration of PM_2.5_ pollutant in all the investigated cities was higher than the WHO standard. In these cities, the annual average concentration of PM_2.5_ was almost 2–4 times higher than the WHO standard. This increase is more due to the reduction of green space and forests, strong wind, low rainfall, heavy dust and the presence of industries and factories and traffic in these cities, which is with the results of the study of Kamarehie et al. and Organization in Bukan city, Hajizadeh et al. In the city of Esfahan et al. Barzeghard in Tabriz et al. Faridi was similar in Tehran [[38], [39], [40]]. Hopke et al. During their research in 10 important cities of Iran, they stated the concentration of PM_2.5_ pollutant between 18 and 105 μg/m^3^, which is 1.8–10.6 times the WHO standard limit. They also observed that in the southern and western parts of Iran due to the phenomenon of dust, the concentration of PM_2.5_ was higher than the rest of the cities [41]. Also, in another study, Ehrampoush Ansari stated that the average annual concentration of PM_2.5_ in Tehran is 3.1 times the WHO permissible limit [42]. Karimi et al. in their research reported the average annual concentration of PM_2.5_ in Ahvaz city for three consecutive years, respectively 5.2, 7.0 and 8.0 times higher than the WHO standard [43]. During a survey conducted in Southwest China, the average daily concentration of PM_2.5_ was 99.5 μg/m^3^, which was higher than the standard [44]. In addition, the average daily concentration of PM_2.5_ was estimated to be 68.95 μg/m^3^ during a research in Ahvaz city, which was higher than the WHO standard [43]. Also, during a study in Tehran during the years 2006–2015, the average annual concentration of PM_2_._5_ 38.8–24.7 μg/m^3^ was observed, which was higher than the WHO limit [41]. During their survey of 22 stations in Tehran, Habibi et al. reported the average annual concentration of PM_2.5_ between 23 and 42 μg/m^3^ [45]. Also, during a research conducted in the city of Tabriz, the highest and lowest annual average of PM_2.5_ was observed as 38.6 and 25.7 μg/m^3^, respectively, which was higher than the permissible threshold [38]. Research results show that people living in these areas have a very high probability of cardiovascular and respiratory diseases [[46], [47], [48], [49], [50]].

**Table 2:** Statistical summary of PM_2.5_ pollutant and meteorological data in this study (2019–2020).

Parameter		Arak	Esfahan	Ahvaz	Tabriz	Shiraz	Karaj	Mashhad
**PM**_**2.5**_**concentration (μg/m**^**3**^)	**Max**	73.92	84.02	170.69	87.45	81.33	84.42	86.49
	**Min**	7.54	11.01	14.94	6.51	0.85	9.91	14.28
	**Mean** ± **S.D**	24.12 ± 6.35	27.93 ± 8.79	43.33 ± 19.23	32.80 ± 17.86	30.85 ± 16.87	26.75 ± 11.51	28.57 ± 9.11
**temperature (**^**◦**^**C)**	**Max**	32.75	33.62	43.52	33.95	35.42	37.06	34.43
	**Min**	−7.66	−2.72	6.04	−9.18	1.57	−5.89	−3.4
	**Mean** ± **S.D**	15.28 ± 10.47	18.85 ± 9.54	29.07 ± 10.08	13.29 ± 10.58	19.12 ± 9.47	16.59 ± 10.66	16.06 ± 9.69
**Relative humidity (%)**	**Max**	90.05	81.98	110.35	94.77	95.76	98.71	96.1
	**Min**	26.4	24.94	56.62	39.65	51.31	41.51	38.51
	**Mean** ± **S.D**	64.45 ± 17.67	55.65 ± 14.45	92.57 ± 14.31	66.14 ± 14.17	78.50 ± 11.61	75.71 ± 13.02	73.70 ± 12.96
**Wind Direction(**^**◦**^**)**	**Max**	54.8	51.95	69.95	56.37	54.69	55.8	55.31
	**Min**	17.6	1.21	30.13	23.3	8.6	11.87	20.86
	**Mean** ± **S.D**	38.93 ± 6.80	27.73 ± 11.67	49.30 ± 7.34	40.65 ± 7.33	31.01 ± 8.47	37.73 ± 9.02	37.74 ± 6.99
**Wind speed(km/h)**	**Max**	100	80.83	83.32	83.04	90.26	95.64	94.67
	**Min**	15.93	0	9.45	14.38	7.1	10.21	12.26
	**Mean** ± **S.D**	46.93 ± 22.67	40.07 ± 16.47	28.32 ± 14	44.89 ± 15.73	26.86 ± 17.14	30.99 ± 14.42	34.24 ± 20.35
**Atmospheric pressure (milli bar)**	**Max**	335	282.91	321.27	272.82	253.28	332.5	270.4
	**Min**	0	110	79.78	70.9	42.06	85.26	72.97
	**Mean** ± **S.D**	171.66 ± 68.17	189.04 ± 40.31	242.42 ± 52.28	142.12 ± 41.58	132.83 ± 42.19	214.40 ± 67.84	133.53 ± 36.48

Several studies have stated; Meteorological parameters affect the concentration of air pollutants [16,39,[51], [52], [53]]. As shown in Tables 2 and in general, the average temperature (^◦^C) has decreased from Ahvaz city to Tabriz city. On the other hand, the average parameters of Relative humidity (%), Wind Direction (◦) and Atmospheric pressure (milli bar) in Ahvaz city have increased significantly compared to other studied cities. Wind speed (km/h) also increased from Shiraz city to Arak.

### 3.2 Health impacts of PM_2.5_ pollutant exposures

Air pollution caused by human activities has significant adverse health effects on people's health [9]. The long-term health effects of exposure to PM_2.5_ pollutants were calculated by AirQ ^+^ software during 2019–2020. These effects included ischemic heart disease (IHD), lung cancer (LC), chronic obstructive pulmonary disease (COPD), stroke, and acute lower respiratory infection (ALRI). Table 3 shows Attributable Proportion (AP), the Number of Attributable Cases (NAC), several Attributable Cases per 100,000 Population at risk (NACPR) and the problems of exposure to PM_2.5_ pollutants. The values of baseline incidence (BI) of ischemic heart disease (IHD), lung cancer (LC), chronic obstructive pulmonary disease (COPD), stroke and acute lower respiratory infection (ALRI) were calculated according to the formula. The maximum and minimum excess cases belonged to 1–332 people and IHD-ALRI disease, respectively (Table 3).

**Table 3:** Health effects attributed to long-term exposure to ambient PM_2.5_ pollutant concentration.

city	Parameter	Health endpoint	Attributable proportion (%)	Excess cases	Attributable cases per 100,000 people
**Arak**	**PM**_**2.5**_	**Natural mortality**	7.39	353	71.43
		**LC mortality**	8.39	4	1.31
		**COPD mortality**	10.43	6	1.95
		**ALRI mortality**	12.21	1	1.62
		**IHD mortality**	14.6	78	22.98
		**Stroke mortality**	14.6	42	12.48
**Esfahan**	**PM**_**2.5**_	**Natural mortality**	8.6	1389	66.88
		**LC mortality**	10.16	13	0.98
		**COPD mortality**	12.37	20	1.56
		**ALRI mortality**	14.63	5	3.49
		**IHD mortality**	15.26	278	19.10
		**Stroke mortality**	15.56	120	8.25
**Ahvaz**	**PM**_**2.5**_	**Natural mortality**	12.32	1092	100.65
		**LC mortality**	16.48	12	1.98
		**COPD mortality**	18.91	9	1.59
		**ALRI mortality**	22.79	3	3.21
		**IHD mortality**	17.29	187	26.81
		**Stroke mortality**	18.54	79	11.40
**Tabriz**	**PM**_**2.5**_	**Natural mortality**	9.92	1573	99.46
		**LC mortality**	12.24	24	2.44
		**COPD mortality**	14.58	28	2.92
		**ALRI mortality**	17.4	4	3.48
		**IHD mortality**	15.98	170	15.69
		**Stroke mortality**	16.6	86	7.97
**Shiraz**	**PM**_**2.5**_	**Natural mortality**	9.42	1291	75.05
		**LC mortality**	11.43	15	1.42
		**COPD mortality**	13.73	19	1.78
		**ALRI mortality**	16.33	3	2.48
		**IHD mortality**	15.71	201	16.81
		**Stroke mortality**	16.2	122	10.17
**Karaj**	**PM**_**2.5**_	**Natural mortality**	8.25	857	45.28
		**LC mortality**	9.63	12	1.01
		**COPD mortality**	11.79	10	0.85
		**ALRI mortality**	13.91	2	1.57
		**IHD mortality**	15.07	258	19.79
		**Stroke mortality**	15.28	63	4.80
**Mashhad**	**PM**_**2.5**_	**Natural mortality**	8.89	2479	83.23
		**LC mortality**	10.6	29	1.78
		**COPD mortality**	12.84	31	1.93
		**ALRI mortality**	15.22	5	1.73
		**IHD mortality**	15.42	332	17.58
		**Stroke mortality**	15.78	197	10.41

In this research, according to what was estimated; respectively, the highest and lowest natural deaths (7.39–12.32), caused by ischemic heart disease (14.6–17.29), lung cancer (8.39–16.48), chronic pulmonary obstruction (10.43–18.91), stroke (14.6–18.54) and Acute lower respiratory infection (12.21–22.79) was related to Ahvaz and Arak cities (Table 3). In general, in this research, the highest and lowest death statistics due to long-term exposure to PM_2.5_ pollutant were 2479 and 353 respectively in the cities of Mashhad and Arak. During the research conducted in Isfahan city by Hajizadeh et al., the highest natural mortality due to exposure to PM_2.5_ pollutant was 21.8% and excess cases of death were estimated at 1440 people. In a study conducted in Tehran by Ansari and Ehrampoush, the highest number of deaths due to long-term exposure to PM_2.5_ was reported as 6710 people [39,42,54]. In another study by Yarahmadi et al. and Hadei et al. in Tehran, they reported the number of deaths due to long-term exposure to PM_2.5_ as 5073 and 4336 respectively [42]. Also, in another review by Karimi et al. the deaths due to long-term exposure to PM_2.5_ in Ahvaz city were estimated by 4061 people [43]. Faridi et al., during his 10-year study in Tehran city, reported the mortality rate of 3755–5895 people due to long-term exposure to PM_2.5_ [39]. During a study by Sicard et al. was conducted in three cities of Iran (Ahvaz Arak and Kermanshah), it was observed that 1914, 611 and 110 people died due to long-term exposure to PM_2.5_ pollution [55]. By Hadei et al., also, during their study in 25 cities of Iran, 13,321 people reported deaths due to long-term exposure to PM_2.5_ [41]. Manojkumar et al. stated the mortality rate of long-term exposure to PM_2.5_ in the cities of Lucknow, Delhi, and Chennai as 26,635, 57,812, and 90,807 respectively [56]. Exposure to PM_2.5_ pollution in the cities of Arak and Karaj compared to other studied cities can be related to the reduction of population in those cities and the duration of exposure [57].

Several studies in different countries have shown that there is a relationship between exposure to air pollution and lung cancer [[58], [59], [60], [61], [62]]. According to what is given in Table 3, the minimum and maximum mortality rates due to lung cancer related to long-term exposure to PM_2.5_ pollutant were 29 and 4 people in Mashhad and Arak cities. During a research conducted in Esfahan, the highest and lowest deaths due to long-term exposure to PM_2.5_ pollutant were reported as 31 and 2 respectively [25]. Also, in a similar study, 7, 75, 24, and 43 deaths were observed in the cities of Tabriz, Ahvaz, Eslamabad, and Rome, respectively, due to long-term exposure to PM_2.5_ [20,38,43,63]. During a survey in Tehran city, the death rate due to long-term contact with PM_2.5_ was stated in 427 cases [54]. During their research in Tehran, Ansari and Ehrampoush stated that the death rate due to lung cancer due to long-term exposure to PM_2.5_ was 27 cases [42]. The results of Faridi et al.'s research in Tehran showed this amount in 83 cases [39]. Also, in another study in Tehran, Yarahmadi et al. reported the number of deaths from lung cancer due to long-term exposure to PM_2.5_ as 142 people [54]. In addition et al. During his study in Tabriz, Barzeghar estimated the number of lung cancer deaths due to long-term exposure to PM_2.5_ to be 5–9 people [38]. The research of Hadei et al. in the city of Ahvaz and 25 cities in Iran showed that 23 and 315 people, respectively, died of lung cancer caused by long-term exposure to PM_2.5_ [41,43]. A three-year study was conducted in 10 Iranian cities and it was observed that 864 people died based on long-term exposure to PM_2.5_ [59,64]. Also, an 8-year study in the city of Taiwan by Hwang et al. found 809–1191 cases of lung cancer mortality due to long-term exposure to PM_2.5_ [65]. During a study by Amoatey et al. in Rome, Italy, 43 people died of lung cancer [20]. In addition, during a study conducted in several large cities in India, the death rate due to lung cancer due to long-term exposure to PM_2.5_ was found to be 358–396 people [56]. The lower mortality rate due to lung cancer in all the studied cities except Mashhad compared to the Ansari and Ehrampoush study in Tehran can be due to the difference in the value of baseline mortalities because the population at risk of the studied cities is lower than the city. It is Tehran. During this research, 48.16% of lung cancer deaths in Ahvaz city were due to long-term exposure to PM_2.5_, which was more than other cities under investigation. In a similar research in the cities of Esfahan and Tehran, Hajizadeh et al. and Ansari and Ehrampoush attributed 17.12% and 17.36% of lung cancer deaths to PM_2.5_, respectively [25]. Also, in another study in Tehran, the mortality rate due to lung cancer due to long-term exposure to PM_2.5_ was stated to be between 13.6% and 19.2% [39,66].

As shown in Table 3, the highest and lowest death rates due to COPD due to long-term exposure to PM_2.5_ pollution were estimated at 31 and 6 people in Mashhad and Arak, respectively. Hajizadeh et al., during their study in Esfahan city, stated the maximum and minimum mortality rates due to COPD as a result of long-term contact with PM_2.5_ pollutant were 22 and 11, respectively [25]. Yarahmadi et al., and Ansari and Ehrampoush, during their studies in Tehran, stated that the death rate due to COPD due to long-term exposure to PM_2.5_ was 158 and 172 people, respectively [42,54]. In another study in Tehran, Faridi et al. reported this value as 123 cases [42]. Also, during the studies of De Marco et al. and Karimi et al. in Ahvaz, respectively 75 and 279 deaths due to COPD were observed as a result of long-term exposure to PM_2.5_ [17,43]. During the research in 25 cities of Iran, the number of deaths and 274 people died from COPD due to long-term exposure to PM_2.5_ [41]. Also, during a study conducted in Tabriz city, it was observed that 67–92 people died from COPD due to long-term exposure to PM_2.5_ lost their lives [38]. In another study conducted in Rome, this amount was reported as 348 people [20]. During another study conducted in Taiwan, this amount was reported as 645 people [67]. In addition, Hwang et al.'s 8-year study in Taiwan city showed that 493-327 people died of COPD due to long-term exposure [38]. Also, Manojkumar & Srimuruganandam reported the death rate of COPD due to long-term exposure to PM_2.5_ in important cities of India as 187–1566 people [56]. In general, during this study, the highest mortality due to COPD due to long-term exposure to PM_2.5_ was 91.18%. During a research conducted by Hajizadeh et al. in Esfahan city; 15.54% of COPD deaths were attributed to long-term exposure to PM_2.5_ pollutants. Also, during the surveys that were conducted in the cities of Mashhad and Tehran; 4.5% and 10.7–15.3% of COPD deaths were related to these causes, respectively. According to the present review, during a study in Tehran, between 10.7% and 15.3% of deaths caused by COPD were attributed to long-term exposure to PM_2.5_ [39,66]. The difference in mortality due to COPD in this study with other studies is probably due to the difference in the value of baseline mortalities and group characteristics.

According to the World Health Organization, the most common cause of death caused by environmental pollutants is IHD [68]. As shown in Table 3, the highest and lowest death rates due to IHD due to long-term exposure to PM_2.5_ pollution were 332 and 78 in the cities of Mashhad and Arak, respectively. The highest and lowest death due to IHD due to long-term exposure to PM_2.5_ during a study conducted in Isfahan city; 285 and 202 people were reported respectively [25]. This amount was 1360 and 2003 cases during a research conducted in the cities of Mashhad and Ahvaz, respectively [43,69]. During the review by Ansari, Ehrampoush and Hadei et al. in Tehran, the mortality rate due to IHD due to long-term exposure to PM_2.5_ was reported as 3797 and 4871 respectively. In another study conducted in Tehran by Faridi et al. was done, this number was reported as 1558 people [42]. Karimi et al. also, during their study in the city of Ahvaz, the death rate due to IHD due to long-term exposure to PM_2.5_ was stated as 2003 cases [43]. Similar to the present study, during a study in Ahvaz by Hadei et al., the number of deaths due to IHD due to long-term exposure to PM_2.5_ was reported as 660 cases [43]. Hadei et al., during their research in 25 Iranian cities, 1536 people stated the death rate due to IHD due to long-term exposure to PM_2.5_ [41]. Also, in a similar study conducted by Hwang et al. in Taiwan city, 1477 people died [65]. In another study conducted by Amoatey et al. in Rome, Italy, the amount of death due to IHD due to long-term exposure to PM_2.5_ was stated as 1189 [20]. Also, during a survey conducted in Taiwan, the death rate due to IHD due to long-term exposure to PM_2.5_ was reported in 2244 cases [67]. In Taiwan, Hwang et al. found the mortality rate of IHD due to long-term exposure to PM_2.5_ to be 1223–1477 people [65]. In general, during this study, the highest mortality due to IHD due to long-term exposure to PM_2.5_ was 29.17%. During the studies conducted in the cities of Isfahan and Tehran, the mortality rate due to IHD due to long-term exposure to PM_2.5_ was observed to be 12.17% and 17.4%, respectively [39,70]. During another similar study in Tehran, it was shown that between 19.8% and 24.1% of deaths caused by IHD were due to long-term exposure to PM_2.5_ pollutants [39,66]. In general, the lower IHD mortality rate in the present study compared to the aforementioned studies can be due to the lower population at risk and the concentration of PM_2.5_ pollutants in the studied cities, which was consistent with the results of Ansari and Ehrampoush's research in Tehran [42].

The relationship between air pollutants and stroke has been the focus of many researchers in recent years and has been proven in many studies [[71], [72], [73], [74], [75], [76], [77]]. During this research, it was observed that the maximum and minimum deaths due to stroke due to long-term exposure to PM_2.5_ pollution in the cities of Mashhad and Arak were 197 and 42, respectively. The results of a study in Esfahan showed that between 55 and 99 people died due to long-term exposure to PM_2.5_ pollution. Also, during the research by Hadei et al. , Ansari and Ehrampoush and Faridi et al. It was done in Tehran; This amount was reported as 1500, 1145 and 604 cases respectively [25]. In another review by Faridi et al. in the city of Tehran, the number of deaths caused by stroke due to long-term exposure to PM_2.5_ was reported by 825 people [42]. Hadei et al. conducted a study in the city of Tehran during the years 2013–2014 and 2014–2015 and reported 2411 and 2396 deaths due to the mentioned cause, respectively [42]. Also, during their research in Ahvaz city and 25 Iranian cities, the number of deaths due to stroke due to long-term exposure to PM_2.5_ was 338 and 963, respectively [41,43]. During a research conducted in Rome, the death rate due to stroke due to long-term exposure to PM_2.5_ was observed in 301 people [20]. Also, during an 8-year study in Taiwan, cases of death due to stroke due to long-term exposure to PM_2.5_ were reported among 1088–2035 people [65]. The difference between the deaths of this study and other studies was probably due to the difference in the value of baseline mortalities. In a recent study, the highest death rate due to stroke due to long-term contact with PM_2.5_ was 54.18%. Similar to the present study, in a study conducted in Tehran, between 24.5% and 36.2% of deaths due to stroke were reported due to long-term exposure to PM_2.5_ [39,66].

Studies have shown that there is a statistically significant relationship between air pollution and death caused by ALRI [78]. So that Darrow et al. in their research, proved that there is a direct relationship between PM_2.5_ pollution and death caused by ALRI [79]. During this study, it was found that the highest and lowest death rates caused by ALRI due to long-term exposure to PM_2.5_ pollutants were 5 and 1 in the cities of Mashhad and Arak, respectively. During the research by Karimi et al. in the city of Ahvaz, the death rate due to ALRI due to long-term exposure to PM_2.5_ was estimated at 30 cases [43]. Ansari and Ehrampoush stated in their research in Tehran that 27 babies died due to long-term exposure to PM_2.5_ due to ALRI [42]. During a similar study by Faridi et al. in Tehran, this amount was reported as 13 people [39]. In addition et al. Barzeghar showed in their study in Tabriz city that they died between 7 and 14 years old due to long-term exposure to PM_2.5_ [38]. Manojkumar & Srimuruganandam, during their study in important Indian cities, stated the death rate due to ALRI due to long-term exposure to PM_2.5_ as 201–1715 people [56]. It seems that one of the important reasons for the difference in the figures was due to the differences in baseline mortalities. During this research, the highest rate of death caused by ALRI due to long-term exposure to PM_2.5_ was observed at 79.22%. Also, during a similar study in Tehran, the death rate due to ALRI due to long-term exposure to PM_2.5_ was reported between 15.0% and 25.2% [39,66].

### 3.3 Ambient PM_2.5_ pollutant concentrations influencing variables

During this research, the effect of variables on PM_2.5_ concentration was evaluated using a time series linear model. Meteorological parameters including temperature, relative humidity, wind speed, wind direction and pressure were received as forecast parameters from the National Meteorological Organization. Table 4 shows the effect of variables on PM_2.5_ concentration. The results show that in Tabriz city, with each unit increase in temperature and wind direction, PM_2.5_ variable increases by 2.32 and 1.19, respectively. The significance of the decrease and the trend of the time series significantly increased over time to 0.36. In the city of Shiraz, for each unit increase in wind speed, the amount of PM_2.5_ significantly decreased by 0.31 and the trend of the time series of PM_2.5_ over time increased significantly by 0.08. In Mashhad city, for each unit increase in relative humidity and wind direction variables, PM_2.5_ increased by 0.56 and decreased by 0.63, respectively. In addition, in the city of esfahan, for each unit of increase in temperature and relative humidity, the amount of PM_2.5_ increased significantly by 0.57 and 0.36, respectively. Also, August had the highest amount of PM_2.5_ among other months. In Ahvaz, the trend of PM_2.5_ changed significantly over time so that with each unit increase in temperature, PM_2.5_ decreased by 0.77. In the month of June, a significant increase of 11.24 was observed in the PM_2.5_ variable. Other variables did not have a significant effect on the target variable, and the amount of PM_2.5_ did not decrease or increase significantly in other months either. In the cities of Arak and Karaj, the linear model of the time series, none of the auxiliary variables had an effect on the trend of the PM_2.5_ time series. The trend of this variable has not undergone significant changes over time, and we did not see any significant increase or decrease in any of the investigated months.

**Table 4:** The influence of variables to ambient PM_2.5_ pollutant concentrations.

	Arak			Esfahan			Ahvaz			Tabriz			Shiraz			Karaj			Mashhad		
	Est	SE	P	Est	SE	P	Est	SE	P	Est	SE	P	Est	SE	P	Est	SE	P	Est	SE	P
**TMP**	−0.35	0.25	0.167	0.57	0.16	0.000	−0.77	0.37	0.042	−2.23	0.44	0.000	0.04	0.20	0.851	0.17	0.40	0.675	−0.09	0.28	0.744
**RH**	−0.05	0.09	0.565	0.36	0.07	0.000	0.10	0.08	0.228	0.56	0.61	0.360	0.01	0.11	0.951	17.06	19.25	0.377	0.56	0.25	0.029
**WD**	0.24	0.16	0.124	0.01	0.12	0.911	−0.67	0.32	0.037	−1.19	0.60	0.050	0.00	0.02	0.968	−0.03	0.25	0.898	−0.63	0.28	0.026
**WS**	−0.02	0.07	0.816	0.00	0.06	0.988	0.13	0.21	0.552	0.62	0.40	0.123	−0.11	0.02	0.000	−16.79	19.27	0.385	0.31	0.17	0.072
**AP**	0.01	0.01	0.314	0.01	0.02	0.511	−0.09	0.04	0.025	−0.04	0.04	0.330	0.00	0.00	0.715	−0.01	0.02	0.545	0.01	0.03	0.678
**trend**	0.01	0.01	0.307	−0.03	0.03	0.377	−0.13	0.05	0.008	0.36	0.13	0.005	0.08	0.00	0.000	−0.01	0.02	0.680	0.11	0.06	0.074
**April**	0.95	1.93	0.624	−0.39	2.08	0.850	−0.04	5.55	0.995	1.60	4.58	0.727	0.21	0.58	0.720	−1.55	3.44	0.653	1.90	2.80	0.497
**May**	−0.52	1.94	0.788	1.62	2.10	0.441	11.24	5.56	0.045	0.34	4.55	0.940	−0.51	0.58	0.378	−0.71	3.45	0.836	−2.95	2.85	0.302
**June**	0.19	1.94	0.921	2.57	2.11	0.226	3.27	5.61	0.561	3.89	4.55	0.395	−0.24	0.58	0.687	−1.57	3.46	0.650	−2.90	2.87	0.313
**July**	1.48	1.97	0.454	6.09	2.10	0.004	8.34	5.62	0.139	−0.01	4.60	0.998	−0.17	0.58	0.775	−2.63	3.51	0.455	−3.69	2.80	0.190
**August**	−0.49	1.95	0.802	0.94	2.10	0.655	4.34	5.61	0.439				−0.43	0.58	0.465	0.59	3.48	0.865			
**September**	2.00	1.95	0.306	0.05	2.10	0.980	−1.16	5.60	0.836				−0.61	0.58	0.296	3.22	3.45	0.352			
**October**	1.98	1.95	0.310																		
**November**	0.21	1.95	0.914																		

RH:Relative humidity; WD: Wind Direction; WS: Wind Speed; AP: Atmospheric pressure; TMP: Temperature; Est: Estimate; SE: Standard error; P: P-value.

## 4 Conclusion

This was a comprehensive research in which the health effects of long-term exposure to PM_2.5_ air pollutants in 7 important and industrial cities of Iran during 2019–2020 were analyzed. In addition, the effect of variables on PM_2.5_ concentration was evaluated with a time series linear model in 7 major cities of Iran. The findings indicate that the annual concentration of PM_2.5_ pollutant in the studied cities is higher than the WHO standard. It seems that the decrease in rainfall, dust storms, loss of vegetation and drought are the main reasons for this increase. The results of the estimation of health effects showed that high concentrations of PM_2.5_ in cities are probably the cause of these adverse health effects. According to the estimate of mortality due to exposure to pollutants, IHD had a wider role in the total morbidity. The difference in the death rate in cities can be related to the difference in the concentration of exposed pollutants, the age of people, the type of culture and lifestyle. It was also observed that meteorological parameters have a very effective role in air quality. It seems that by implementing appropriate control measures such as removing old cars and replacing them with new cars, encouraging people to increase the use of the public transportation system, improving the fuel quality of motor vehicles, using more electric and hybrid vehicles, Heavy taxes from industries and power plants with high pollutant emissions will significantly reduce economic and health losses.

## Funding

This research did not receive any specific grant from funding agencies in the public, commercial, or not-for-profit sectors.

## Data availability statement

Data included in article/supplementary material/referenced in article.

## Authorship statement

The authors declare no competing financial interests or personal relationships that could influence this work.

## Additional information

Supplementary content related to this article has been published online.

## Ethical approval

This project has been registered in Iran University of Medical Sciences with the code of ethics of IR.IUMS.REC.1400.006.

## CRediT authorship contribution statement

**Zahra Kazemi:** Conceptualization, Methodology, Investigation, Formal analysis, Writing – original draft. **Ahmad Jonidi Jafari:** Methodology, Writing – review & editing, Investigation. **Mahdi Farzadkia:** Methodology, Writing – review & editing, Investigation. **Payam Amini:** Methodology, Writing – review & editing, Project administration. **Majid Kermani:** Methodology, Writing – review & editing, Supervision, Funding acquisition.

## Declaration of competing interest

The authors declare no conflict of interest.

## Figures and Tables

**figs1:**
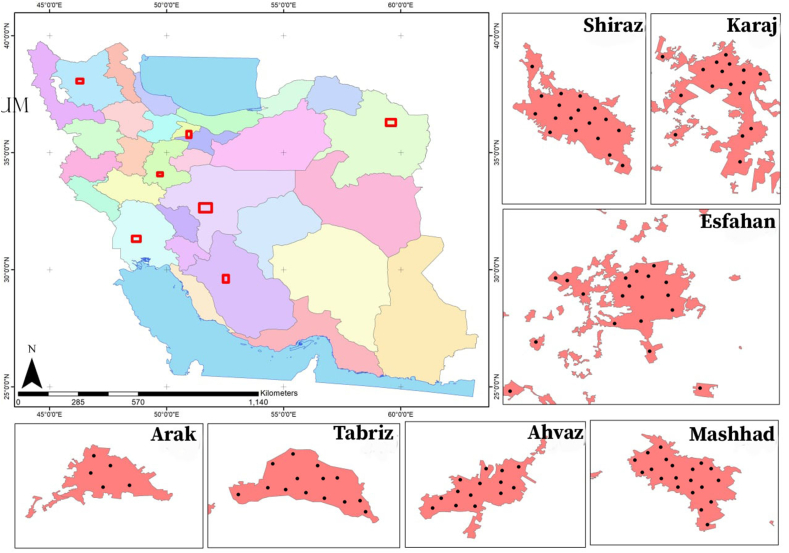


## Abbreviations

WHO: World Health Organization
COPD: Chronic obstructive pulmonary disease
IHD: Ischemic Heart Disease
BI: Basline Incidence
RR: Relative Risk
PM_2.5_: Particulate Matter Less than 2.5 μm
PM_10_: Particulate Matter Less than 10 μm
NO_2_: Nitrogen dioxide
O_3_: Ozone
ALRI: Acute Lower Respiratory Infection
LC: Lung Cancer
